# Factors associated with anxiety-related symptoms in children and adolescents during COVID-19 in Oman: a cross-sectional study

**DOI:** 10.1038/s41598-024-59769-y

**Published:** 2024-04-29

**Authors:** Muna Al-Shekaili, Salim Al-Huseini, Yahya Al-Kalbani, Hassan Mirza, Moon Fai Chan, Walid Hassan, Fatma Al-Sulimani, Ghaniya Saif Al-Ghafri, Hanan Saif Al-Sumri, Ahmed Bait Amer, Aishwarya Ganesh, Muna Al-Saadoon, Samir Al-Adawi

**Affiliations:** 1 Al Massarah Hospital, Ministry of Health, Muscat, Oman; 2 Department of Behavioural Medicine, College of Medicine & Health Sciences, Sultan Qaboos University, Muscat 123, Oman; 3 Department of Family Medicine and Public Health, College of Medicine & Health Sciences, Sultan Qaboos University, Muscat 123, Oman; 4 Directorate General of Health Services, Al Dhahira Governorate, Ibri Regional Hospital, Ministry of Health, Ibri, Oman; 5 Directorate General of Nursing Affairs, Ministry of Health, Muscat, Oman; 6 Department of Child Health, College of Medicine & Health Sciences, Sultan Qaboos University, Muscat 123, Oman; 7 The Okasha Institute of Psychiatry, Ain Shams University, El-Demerdash, El Abbassia, Cairo 11657 Egypt

**Keywords:** Psychological distress, COVID-19, Anxiety, Anxiety-related symptoms, Child and adolescent, Oman, SCARED, Human behaviour, Psychology

## Abstract

Reports from different parts of the world suggest that the COVID-19 pandemic and the resultant lockdown and social distancing measures have heralded unprecedented mental health challenges among children and adolescents. To date, there is a dearth of studies emerging from the Arabian Gulf, where the majority of its population are children and adolescents. The study aims to examine the prevalence of anxiety-related symptoms and their covariates among children and adolescents in an Arabian Gulf country, Oman. This is a cross-sectional analytic study carried out over two weeks (1st to 15th of August 2020) during the COVID-19 pandemic across Oman. Parents were asked to complete the online survey, which consisted of the parent version of the *Screen for Child Anxiety Related Emotional Disorders* (SCARED) instrument and questions regarding basic socio-demographic information. Logistic regression was used to identify the contributing variables associated with anxiety-related symptoms. A total of 790 valid responses were received. Among the 790 children, 33.3% (n = 263) were diagnosed with anxiety-related symptoms by the SCARED instrument. Logistic regression analysis suggested that anxiety-related symptoms in children and adolescents were significantly associated with three demographic variables. The model shows that children with divorced or separated parents were 1.9 times more likely to have anxiety-related symptoms than children of married couples (OR = 1.93, p = 0.035). Children living in families with an income below USD 1000/month, were 1.8 times more likely to have anxiety-related symptoms than a family with an income of USD 4000/month (OR = 1.833, p = 0.018). Children in grades 3–6 were 1.8 times more likely to have anxiety-related symptoms than those in grades 1–2 (OR = 1.79, p = 0.024). Anxiety-related symptoms are common among Omani children and adolescents. They are more likely to be reported in middle scholastic grade levels and children from families with marital discord and low socioeconomic status. It is not clear whether the presently observed rates of anxiety exceed the prevalence that would have been observed prior to the COVID-19 pandemic. More studies are therefore warranted using children and adolescents' self-reported scales.

## Introduction

A novel strain of coronavirus, entitled ‘Severe Acute Respiratory Syndrome Coronavirus 2 (SARS-CoV-2), was first identified to affect human beings in December 2019, and subsequently reported to trigger respiratory illness. SARS-CoV-2 comes to be known as coronavirus disease 2019, or COVID-19^1^. The World Health Organization (WHO) declared the outbreak a public health emergency of international concern in January 2020, and a pandemic on March 11, 2020^2^. As of April 1, 2021, more than 2 million deaths worldwide were attributed to COVID-19^3^.

While vaccines continued to be dispensed in different parts of the world, preventive measures are widely considered to be the most effective means to curb the rising tide of COVID-19, to date^4^. Social and physical distancing, including quarantines and travel restrictions, have curbed all forms of social interactions in public settings such as educational institutions, workplaces, playgrounds, theatres, and marketplaces^4^. These measures have been postulated to harm the social and emotional functioning of children and adolescents^5^. Overall, emerging data suggests that children and adolescents are adversely affected by the COVID-19 pandemic, and there is a rising tide of poor mental health outcomes in them^6^. This is congruent with other literature that observes that during periods of economic and social instability, there is a high risk of disruption of family functioning, parental stress, and caustic relationships in households^7^. As a result, situations arise that echo the proverb *“when elephants fight, it is the grass that suffers”*, which implies that during periods of family disruption, it is the weak or ‘dependant’ individuals that typically suffer. Meherali et al.^8^ conducted a systematic review and thematically analyzed the impact of the pandemic on children and adolescents' mental health. The authors concluded that this age group has high-risk factors for poor mental health outcomes that are likely to persist even after lockdown measures are relaxed. The conclusion is consistent with vast empirical data suggesting poor mental health among adults often has roots in childhood adversity^9^.

Orgilés et al.^10^ surveyed 1143 parents of Italian and Spanish children aged 3 to 18 years old for the emotional impact of the quarantine on children. This study indicated that approximately 86% of the parents noted changes in the functioning of their children during the quarantine, displaying cognitive symptoms as well as having a spectrum of internalizing and externalizing behavior problems. In the UK, Groarke et al.^11^ examined the prevalence of loneliness during the pandemic. The study was conducted among the general population, and the data revealed that the observed prevalence rate of loneliness was more prevalent among the younger age group.

Even before the pandemic, there was evidence to suggest that forced isolation is associated with increased risk factors for poor mental health outcomes. Loades et al.^12^ conducted a systematic review of 63 studies on the impact of forced social isolation on the mental health of children and adolescents. This critical appraisal, which spans from the year 1946 to 2020, suggested that children and adolescents in enforced isolation are likely to manifest higher rates of affective symptoms. Supporting this conclusion, Magson et al.^13^ reported that adolescents in Australia tend to experience pronounced elevated scores in indices of affective symptoms during the COVID-19 pandemic. Of note, this longitudinal study suggests that there is an increment of such distress as adolescents continued to dwell in confinement. In the Chinese population, Zhang et al.^14^ conducted longitudinal studies on the presence of affective symptoms and suicidal behavior among Chinese children and adolescents prior to the COVID-19 outbreak and after the lockdown was lifted. The authors reported poor mental health outcomes persisting after the lockdown. These studies all strongly imply that lockdowns tend to toil on the mental health integrity of younger age. Thus, careful attention to the identification and mitigation of poor mental health outcomes during formative years has the potential to reduce many adverse health and social outcomes that have the potential to persist in adulthood^9^.

While there is robust evidence of the COVID-19 pandemic tending to increase loneliness and lead to poor mental health outcomes among children and adolescents in other parts of the world to date^12^, there is a dearth of studies from the Arabian Gulf countries. Arabian Gulf countries such as Oman. In demographic parlance, Oman is currently in the midst of the second stage of demographic transition, implying a high birth rate and predominance of the younger age group. It currently has a pyramidal population structure with a “youth bulge”^15^.

The global pandemic of COVID-19 first affected Oman on February 24, 2020, when two cases of COVID-19 were confirmed in the country^16^. During the time when this study was conducted, there were 73,791 positive cases of COVID-19 and approximately 359 deaths due to COVID-19 complications. The spike in cases led to the imposition of nationwide lockdowns. On May 7, 2020, the Ministry of Education decreed to close all educational centers. There was a period when there were intermittent allowances for secondary school students to attend classes, but the increasing number of cases eventually resulted in only ‘tele-education’ or e-learning being allowed.

This study has been conducted to explore whether the prevailing enforced social isolation during the COVID-19 pandemic has adversely impacted the psychological functioning of children and adolescents in Oman, manifesting as anxiety-related symptoms. The specific aims of this study were to examine the prevalence of anxiety-related symptoms in this age group, as well as the contributing variables associated with them.

## Methodology

### Participants

This cohort study is part of a wider study exploring mental health factors associated with early adversity among children in the Gulf Cooperation Council (GCC). The GCC is the political and economic alliance of six countries located in the Arabian Peninsula, namely Bahrain, Kuwait, Oman, Qatar, Saudi Arabia, and the United Arab Emirates. It was established in 1981 to promote economic, social, and political cooperation among its member states. The original study included data from three GCC countries: Oman, Saudi Arabia, and Kuwait. The present study reports on the cohort from Oman.

This is a web-based cross-sectional analytic study, carried out over 2 weeks between the 1st and 15th of August, 2020. The present study recruited consenting parents of children and adolescents (aged 8–18). Oman has two types of educational systems –private and public (or ‘governmental’), and students from both were enrolled in the study. The Ministry of Education finances the latter education system, providing services free of charge up to the end of secondary education (grade 12). With the majority of Oman being under 20 years of age, national educational initiatives have grown exponentially in recent years, from three formal schools with 900 students in the previous decades to 1053 public schools currently. In urban areas, there has been concurrent growth of private schools, which provide a bilingual stream of education.

Inclusion criteria consisted of the parents of children between the ages of 8 to 18 residing in Oman during the pandemic. Exclusion criteria consisted of the parents of children in schools catering solely to children and adolescents with cognitive, social or learning disabilities. In Oman, children with severe forms of such disabilities often attend specialized, accessible schools. However, children with milder forms of learning disabilities are generally accommodated in mainstream education. The protocol of the present study was not equipped to exclude such children and adolescents. Children with a diagnosis or history of behavioral and/or mental disorders prior to the onset of the pandemic were another exclusion criterion that was considered.

### Procedure

After obtaining ethical approval from the Institutional Research Board (IRB) ( Centre of Studies and Research, Ministry of Health), the Ministry of Education overseeing public and private school education was approached to obtain official permission and cooperation to conduct the survey. Education in Oman is universal and free throughout all three scholastic levels—primary, middle/intermediate, and secondary. The Ministry of Education in Oman has 1500 public schools across the country. At the time when this study was conducted, there were 2046 schools officially registered in the 11 *muhafazah,* or administrative regions of Oman^17^, with a total number of 843,598 students (with an average of 27 students per class)^18^. The majority of the population lives in the northern part of the country, within the capital city of Muscat and the adjacent satellite towns known as the Batinah Coast. This region stretches from the border with the United Arab Emirates in the west to the Musandam Peninsula in the north. Since approximately 85% of the population reside in the capital and the Batinah Coast^19^, the rest of the population is in the south, which is separated from the rest of the population in the north by the Empty Quarter, also known as the *Rub' al Khali*. The Ministry of Education, according to its internal auditing standards, have established representative catchment among enrolled students of both the public and private schools. We took advantage of the pre-existing system to approach the representative schools. The competent authorities for the schools in different parts of the country were contacted to cooperate in conducting this survey via the Ministry of Education. Attempts were made to recruit participants from all 11 *muhafazah*. One school—private or public—was randomly sampled from each *muhafazah*. The sample size and selection criteria were chosen to ensure that the sample is representative of the entire school-going population of Oman.

To conduct the study, the concerned school authorities that responded to the investigators' initial inquiry were contacted through e-mail, WhatsApp, or phone calls to elaborate on the purposes of the survey. Google forms were then sent through email and WhatsApp to the principals of the targeted schools.

### Sample size calculation

We based our sample size on a previous study by Al-Salmani et al.^20^ that reported the prevalence of depressive symptoms in attendees of urban primary healthcare centers in Oman to be 8.1%, using the patient health questionnaire (PHQ). By using online software (EPITOOLS, 2020)^21^, with a 95% confidence interval, a 2.5% margin of error, and with unknown population size, the required sample size for the survey was calculated to be 458.

### Data collection

Parents who fulfilled the inclusion and exclusion criteria were requested to complete the study survey. In the attached description of the study, participants were provided details about the study and were explicitly informed that they were at liberty to terminate their participation in the study at any time if they so wish, without providing any justifications to the investigators. The survey was anonymous, and the confidentiality of the personal information collected was assured. All participants were informed that by accessing the first page of the survey, they were expressing their approval and consent to participate in the study. Questionnaires were available both in English and in Arabic. Google forms were used to generate the questionnaires and links for parents were distributed using email and social media platforms. A total of 1500 forms were dispensed to potential participants and a total of 790 valid responses were received. This gave a response rate of 52.7% (790/1500).

### Outcome measure

#### The screen for child anxiety-related emotional disorders

The Screen for Child Anxiety-Related Emotional Disorders (SCARED) is a 41-item Likert scale designed to tap into the severity of child and adolescent anxiety-related symptoms derived from Generalized Anxiety, Separation Anxiety Disorder, Social Anxiety Disorder, School Phobia, as well as panic and somatic distress often accompanying such anxiety disorders. The respondent is supposed to refer to the child’s or adolescent’s anxiety-related symptoms over the past three months^22^. Responses are recorded on a 3-point Likert scale (0 = “Not True, or Hardly Ever True”, 1 = “Somewhat True or Sometimes True” or 3 = “Very True or Often True”).

There are versions of the SCARED for both the child/adolescent and the parent to report through. Due to logistical and ethical considerations, we have utilized the parental version of SCARED, which we have refer to as P-SCARED-41. The rationale behind using the parental version is the ongoing COVID-19 pandemic, which posed difficulties to the investigators attempts to contact both children and their parents or guardians. To use the child version, it would have been necessary to also contact parents or guardians to provide consent for their ward (between the ages of 0 to 18 years) to participate in online research. For this reason, only parents were approached to fill out the research instrument.

P-SCARED-41, which is designed for parent-proxy reporting for their child, has advantages as well as disadvantages. On one hand, potential inaccuracies in reporting are possible due to the parent’s perceptions, biases and flawed understanding of the child's emotions. Thus, the data obtained through P-SCARED-41 may not fully capture the complexity or nuances of the child's experiences. These limitations were difficult to overcome during the pandemic, when it was only possible to collect data online.

Several studies have compared the reliability of P-SCARED-41 with the child self-reported versions. The results have been mixed, with some studies reporting high levels of agreement between parent-proxy and child self-report scores, while others have found low levels of agreement^22–24^. A meta-analysis by Runyon et al.^25^ indicated moderate to large agreement between the two. The level of agreement may vary depending on the the age of the child, the specific anxiety symptoms being assessed, and other factors.

P-SCARED-41 has been translated into various languages and their psychometric properties have been reported to be adequate^26^, including the presently used Arabic version^27,28^. English is also widely spoken in Oman, and to accommodate non-Arabic-speaking parents, the English version of P-SCARED-41 was also included. Both versions of the SCARED questionnaire are publicly available^29^.

The internal consistency of the P-SCARED-41 questionnaire used in the current study was found to be adequate (Cronbach's α = 0.89). A reported questionnaire yielding a total score of 25 or above is considered suggestive of child anxiety-related disorders^30^. Hence, this cut-off point was used in this study to indicate the presence of child and adolescent anxiety-related symptoms. There is also a cut-off for each subscale (Panic Disorder or Significant Somatic Symptoms = 7 cut-off, Generalized Anxiety Disorder = 9 cut-off, Separation Anxiety Disorder = 5 cutoff, Social Anxiety Disorder = 8 cut-off, and School Avoidance = 3 cut-off). For statistical stability, only composite scores (labelled as ‘child anxiety-related symptoms’) are presented in the present study.

In addition to the SCARED questionnaire, the Google form consisted of questions devoted to collecting detailed sociodemographic data of the children and their parents, as shown in Table 1. Questions focusing on certain challenges faced by them owing to the unique circumstances of the COVID-19 pandemic and the resultant lockdown were also asked, and the data collected is presented in Table 1.

**Table 1:** Characteristics of the parents of children and adolescents (aged 8- 18) (n = 790).

Demographic	n (%)
Respondent	
Father of the child	163 (20.6)
Mother of the child (ref)	627 (79.4)
Marital status of the parent	
Divorced/Separated	47 (5.9)
Married (ref)	743 (94.1)
Age (Years) of the respondent	
< = 34	167 (21.1)
35–44	456 (57.7)
45 + (ref)	167 (21.2)
Educational level—Father	
High school or below	353 (44.7)
College degree	250 (31.6)
Post-graduated (ref)	187 (23.7)
Educational level—Mother	
High school or below	437 (55.3)
College degree	185 (23.4)
Post-graduated (ref)	168 (21.3)
Family income/month (USD)	
< 1000	134 (17.0)
1000–1999	292 (37.0)
2000–3999	177 (22.4)
4000 + (ref)	187 (23.6)
Current working status	
Not work (unpaid leave/terminated)	48 (6.1)
Working (ref)	742 (93.9)
Paid leave	160 (21.6)
Working fewer hours	129 (17.4)
Working regular hours	415 (55.9)
Working more hours	38 (5.1)
Family history of mental illness	
Yes	27 (3.4)
No	763 (92.6)
Grade level of the child	
7–12	115 (14.6)
3–6	483 (61.1)
1–2 (ref)	192 (24.3)
Age (Years) of the child	
8–11	730 (92.4)
12–18 (ref)	60 (7.6)
Gender of the child	
Girl	387 (49.0)
Boy (ref)	403 (51.0)
SCARED-41	
Anxiety disorder (total score > = 25, Yes)	263 (33.3)
Panic disorder (sub-total score > = 7, Yes)	220 (27.8)
Generalized anxiety disorder (sub-total score > = 9, Yes)	129 (16.3)
Separation anxiety disorder (sub-total score > = 5, yes)	168 (21.3)
Social anxiety disorder (sub-total score > = 8, yes)	117 (14.8)
Significant school avoidance (sub-total score > = 3, yes)	392 (49.6)

*SCARED-41* Screen for Child Anxiety-Related Emotional Disorders scale.

### Statistical analysis

Data was analyzed by IBM Statistical Package for Social Sciences or SPSS (IBM SPSS23.0 NY, USA), and the results of those who were identified as having anxiety symptoms (SCARED score >  = 25) or identified as without anxiety symptoms (SCARED score < 25) were analyzed using descriptive statistics. Univariate analysis was conducted, and demographic variables were evaluated with the chi-square test and odds ratio (OR) to explore the association between the two groups. Next, multivariate analysis was conducted using a logistic regression model, with child anxiety-related symptoms as the dependent variable, and those variables with p < 0.05 in the univariate analysis as the independent variables, and concurrently adjusted by each other. This analysis helped address the research aim to identify the contributing variables associated with child anxiety-related symptoms. A p-value of < 0.05 was considered statistically significant.

### Ethical approval

This study was approved by the Centre of Studies and Research at the Ministry of Health (MoH/CSR/19/10009). Informed and signed consent was obtained from the parents or guardians of the children. The study was conducted following the Declaration of Helsinki with regard to ethical human research, including confidentiality, privacy, and data management.

### Ethics approval and consent to participate

This study was approved by the Institutional Review Board (MoH/CSR/19/10009) under the auspice of the Ministry of Health. All participants were provided written informed consent to participate. An overseeing mental health professional declared all participants to be capable of providing ethical consent for their participation. Informed and signed consent was obtained from the parents or guardians of the children. The study was carried out under the Code of Ethics of the World Medical Association (Declaration of Helsinki) for human experiments.

## Results

A total of 790 participants fulfilled the inclusion criteria. Table 1 presents the results for both the parent’s (or ‘Respondent’) and children’s demographic variables. Out of 790 children, 33.3% (n = 263) were diagnosed with anxiety-related symptoms based on the P-SCARED-41 scale. Specifically, 220 (27.8%), 129 (16.3%), 168 (21.3%), 117 (14.8%), and 392 (49.6%) were diagnosed with panic disorder, generalized anxiety disorder, separation anxiety disorder, social anxiety disorder, and significant school avoidance disorder, respectively. More mothers participated in the study (n = 627, 79.4%) than fathers (20.6%). The majority of the respondents were married (n = 743, 94.1%), and more than 70% (n = 623) were ≥ 35 years old. More than half (n = 426, 54.0%) of them had an income below USD 1, 999 per month, and 93.9% (n = 742) of them were employed during the COVID-19 pandemic. More than 70.0% (n = 627) of the respondent mothers had a high school level or lower education level (n = 437, 55.3%). In contrast, a majority of the respondent fathers had a college degree or higher education level (n = 437, 55.3%). The majority of the children of the participants were aged 8–11 years old (n = 730, 92.4%) with more than 61% (n = 483) studying in grades 3–6. The proportion of girls (49.0%) and boys (51.0%) were almost equal.

Table 2 shows univariate and multivariate (logistic regression) analysis for sociodemographic data and its association with the child's anxiety. In the univariate analysis, a significant association was found between anxiety-related symptoms and the marital status of the parent (‘not married’ vs. ‘married’, p = 0.043), educational level of the mother (‘college degree’ vs. ‘post-graduate’, p = 0.037), family income per month (‘ < 1000’ vs. ‘4000’, p = 0.002; 1000–1999 vs. 4000, p = 0.021), and the child’s grade level at school (‘7–12’ vs. ‘1–2’, p = 0.014; ‘3–6’ vs. ‘1–2’, p = 0.009). The multivariate logistic analysis suggested a significant association of three demographic variables with the anxiety-related symptoms. The model showed that children whose parents were divorced or separated were 1.9 times (OR = 1.93, p = 0.035) more likely to have anxiety-related symptoms than children who had parents who were married. Children whose family income was below USD 1000 per month were 1.8 times (OR = 1.83, p = 0.018) more likely to have anxiety-related symptoms than those with a family income of USD 4000 per month. Children in grades 3–6 were 1.8 times (OR = 1.79, p = 0.024) more prone to have anxiety-related symptoms than those in grades 1–2.

**Table 2:** Univariate and multivariate (logistic regression) analysis for child anxiety-related symptoms in association of demographic factors.

Demographic	Anxiety-related symptoms		Univariate analysis		Multivariate analysis^	
	Yes (n = 263)	No (n = 527)				
	n (%)	n (%)	OR	p-value	OR	p-value
Respondent						
Father of the child	56 (21.3)	107 (20.3)	1.062	0.746		
Mother of the child (ref)	207 (78.7)	420 (79.7)				
Marital status of the parent						
Divorced/Separated	22 (8.4)	25 (4.7)	1.833	0.043*	1.931	0.035*
Married (ref)	241 (91.6)	502 (95.3)				
Age (Years) of the respondent						
< = 34	69 (26.2)	98 (18.6)	1.557	0.053		
35–44	142 (54.0)	314 (59.6)	0.999	0.999		
45 + (ref)	52 (19.8)	115 (21.8)				
Educational level—Father						
High school or below	118 (44.8)	235 (44.6)	1.117	0.569		
College degree	87 (33.1)	163 (30.9)	1.187	0.406		
Post-graduated (ref)	58 (22.1)	129 (24.5)				
Educational level—Mother						
High school or below	141 (53.6)	296 (56.2)	1.263	0.244	1.15	0.506
College degree	76 (28.9)	109 (20.7)	1.849	0.037*	0.728	0.101
Post-graduated (ref)	46 (17.5)	122 (23.1)				
Family income/month (USD)						
< 1000	57 (21.7)	77 (14.6)	2.085	0.002*	1.833	0.018*
1000–1999	106 (40.3)	186 (35.3)	1.605	0.021*	1.504	0.115
2000–3999	51 (19.4)	126 (23.9)	1.14	0.577	1.132	0.582
4000 + (ref)	49 (18.6)	138 (26.2)				
Current working status						
Not work (unpaid leave/terminated)	18 (6.8)	30 (5.7)	1.217	0.523		
Working (ref)	245 (93.2)	497 (94.3)				
Paid leave	59 (24.1)	101 (20.3)	5.892	0.117		
Working less hours	38 (15.5)	91 (18.3)				
Working regular hours	130 (53.1)	285 (57.4)				
Working more hours	18 (7.3)	20 (4.0)				
Family history of mental illness						
Yes	13 (4.9)	14 (2.7)	1.905	0.096		
No	250 (95.1)	513 (97.3)				
Grade level of the child						
7–12	44 (16.7)	71 (13.5)	1.859	0.014*	1.097	0.073
3–6	171 (65.0)	312 (59.2)	1.644	0.009*	1.793	0.024*
1–2 (ref)	48 (18.3)	144 (27.3)				
Age (Years) of the child						
8–11	247 (93.9)	483 (91.7)	1.406	0.257		
12–18 (ref)	16 (6.1)	44 (8.3)				
Gender of the child						
Girl	128 (48.7)	259 (49.1)	0.981	0.899		
Boy (ref)	135 (51.3)	268 (50.9)				

*Ref* reference point, Chi-square test,* OR* odds ratio. *, sig., p < 0.05; SCARED (Screen for Child Anxiety Related Emotional Disorders): Yes: 25 +, No: < 25.

^Logistic (Enter); Hosmer and Lemeshow Test, χ^2^ = 6.875, p = 0.442; Sensitivity = 61.6%, Specificity = 54.3%, overall predicting power = 56.7%.

## Discussion

Studies examining the prevalence of child and adolescent mental health problems have only recently begun to emerge from the Arabian Gulf countries such as Oman. The prevalence rates of children and adolescents in Oman with disordered eating habits is an estimated 9.5%^31^, those with autism spectrum disorders in the range of 1.4–20.35/10,000^32^, and those with ADHD an estimated 8.8%^33^. Other children and adolescents' mental health problems, including but not limited to social phobia, mood disorders, learning disorders, and other adverse childhood experiences have also been reported^34^. In Uganda, it has been widely speculated that among many varied child mental health problems, anxiety appears to be most prevalent with a figure ranging from 10 to 20% of children and adolescents exhibiting anxiety-related symptoms^35^. In the USA, using a face-to-face survey of 10123 Americans (ages between 13 and 18 years), Burstein et al.^36,37^ reported that around 3% and 9% of the participants have core features of generalized anxiety disorder. In a recent meta-analysis of six Arabian Gulf countries by Chan et al.^38^, out of the accrued 33 studies that fulfilled the study criteria, the pooled prevalence of anxiety symptoms ranged from 17.27 to 57.04%. This critical appraisal covered the pre-pandemic era. It is not clear how the pandemic and all of the resulting tribulations have affected the child and adolescent population in Oman.

The COVID-19 pandemic has resulted in children and adolescents experiencing unprecedented interruptions to their daily lives, which are likely to precipitate poor mental health outcomes^8^. Studies carried out in different parts of the world during the pandemic strongly suggest this outcome, with the assumption that enforced social isolation is adversely impacting children's psychological functioning which is, in turn, conventionally manifesting as anxiety-related symptoms^6–8,10,11^. Therefore, the present study has been conducted to investigate the prevalence rate of anxiety-related symptoms in the children and adolescents of Oman during the COVID-19 pandemic.

Prior to the COVID-19 pandemic, the international prevalence of child and adolescent mental health problems constituted 13.4% of the sample of the 27 countries surveyed, with those afflicted with anxiety-related symptoms constituting 6.5% of the pooled sample^39^. In the United States, Hawes et al.^40^ suggested that the frequency of anxiety-related symptoms tends to strongly hinge on whether the region is experiencing a period of peak infection rates and having resultant lockdowns. This implies that anxiety-related symptoms are situational. Compared to the global rate before the COVID-19 pandemic, the present rate appears to be higher than the pooled prevalence of 13.4%. There are various instruments available to solicit the presence of anxiety-related symptoms, and the reported prevalence rates may be impacted by the instrument used to study them. Studies that specifically employ the SCARED questionnaire also may have results that are likely to vary when using the self-rated child version or when answering by proxy using the parent version. Therefore, comparisons between various studies would likely echo the metaphor of *‘comparing apples and oranges’*, if this factor is not taken into consideration.

Ravens-Siebereret al.^41^ conducted a nationally representative study (7- to 17-year-old children and adolescents) in Germany employing the child self-rated SCARED questionnaire, reporting a 24.1% prevalence of children who have reached the threshold for case-ness of anxiety-related symptoms. In Iran, among adolescents with hearing loss during the COVID-19 pandemic, Ariapooran and Khezeli^42^ reported 37.5% of the study participants had anxiety-related symptoms. The present study in Oman suggests that 33.3% (n = 263) fulfilled the criteria for anxiety-related symptoms, as defined by the SCARED questionnaire. Among subtypes of anxiety-related symptoms, school avoidance was exhibited among 49.6% of the participants. The second most endorsed anxiety-related symptoms was panic disorder (27.8%), followed by separation anxiety disorder (21.3%), generalized anxiety disorder (16.3%) and social anxiety disorder (14.8%) was the least endorsed. Previous studies on the frequency of case-ness of social anxiety disorder were 45.9% among the adult population in Oman and 36.6% among the school-going population^43^. It is likely that the frequency of anxiety-related symptoms tends to fluctuate in complex ways due to situational and sociocultural factors^44^.

In several non-western societies, sociocultural patterning tends to predominantly usher children into the collective mindset. There is evidence to suggest a tendency to lean towards a ‘collective’ social orientation, which is in contrast to the ‘individualistic’ social orientation commonly found in Western countries. To preserve social harmony, according to Al Asmi et al.^45^, “*such a society tends to discourage the expression of emotion and therefore relegates emotional distress. Instead, distress is expressed in somatic terms, a feat that is orthogonal to what may constitute emotional distress in the psychiatric nomenclature…*” (p.6). To extrapolate from such a view in the context of anxiety symptoms, being marked with somatic distress, anxiety symptoms are likely to be recognized and acknowledged by the parents of the children in Oman. In contrast, this view would imply that emotional distress, since it is stigmatized, is unlikely to be acknowledged. With this background, it is, therefore, safe to speculate that anxiety symptoms were highly endorsed by the parents because their manifestation does not contravene socio-cultural teachings. In the regions in the global south with the bulk of its population being children and adolescents, studies are needed to explore the interplay between cultural patterning and odium to distress.

As is often the case, mental distresses tend to be intimately linked to socio-demographic factors. To address such a link, the related aim of this study is to comprehensively examine which demographic factors unique to this period contribute to the development of anxiety symptoms solicited by the *Screen for Child Anxiety Related Emotional Disorders* (SCARED). As shown in Table 1, this study accrued various sociodemographic factors, including the type of family caregiver (mother or father). The majority of respondents to the survey were mothers rather than fathers, constituting 79.4% of the sample. Other sociodemographics sought from the parent included marital status of the parent, educational level of both parents, family income, current working status, family history of mental illness. For the children and adolescents, the grade age and gender of the child were sought. Both univariate and multivariate analysis was used to examine the associated factors with symptoms of anxiety. As shown in Table 2, socio-demographic factors significantly associated with anxiety symptoms include having divorced or separated parents, a family income below 1000 US dollars, and studying in scholastic grade levels between 3 to 6. These significant associated factors are recapitulated within the background literature below in tandem.

First, divorce was significant in the regression analysis. Recent divorce or separation may be likely to cause an acute stress reaction that may contribute to the chronicity of poor mental health outcomes. This study could not determine whether the divorce coincided with the outbreak of the pandemic or whether it preceded the pandemic. Current data shows that most of the respondents were married and were 35 years or older. Divorced or separated parents consisted of 8.4% of participants whose children were significantly associated with higher SCARED scores. It is well known that regardless of age, gender, and culture, children of divorced parents experience tend to succumb to poor coping mechanisms, and this could culminate in an increased risk of developing poor mental health outcomes^46^. Future studies, if the present study withstands further scrutiny, should define the types of separation or divorce and whether they occurred during the pandemic. It should also be critical to unravel whether the divorce/separation is recent or not. In addition to divorce or separation, it has been widely documented that required quarantine and social isolation have significantly reduced the opportunity to escape from caustic home environments. As a result, according to Lebow^47^, *“couples fall into angry exchanges without resolution or into patterns of demand-withdrawal, which may degenerate into protracted high conflict and sometimes domestic violence. In parallel, social interactions and social support that might mitigate tensions are decreased”* (p. 968). This echoes the proverb *“When the elephants fight, the grass gets trampled*”^48^. Studies are needed on whether periods of quarantine and isolation, unstable family dynamics, and separation from a parental figure can be detrimental for children requiring greater amounts of emotional support.

Second, according to regression analysis, family income was significant. Some studies suggest that the pandemic disproportionately affects children who are at a socio-economic disadvantage^8,49^. In support of such a view, the present study showed that there is an association with the higher SCARED score in children and adolescents of families with low socio-economic status (specifically, those with a family income below 1999 USD per month). Low-income families are facing greater degrees of financial instability during the pandemic, with enforced lockdowns and curfews making it challenging for parents to meet the demands of dependent family members and children. The pandemic has also caused economic instability in the country which, in turn, is causing a lack of job security and fluctuating incomes^50^. The resultant increased psychological burden on parents can simultaneously affect their children as well, which is a phenomenon that has been widely documented in the setting of the COVID-19 pandemic, including countries like Italy, Germany, and China^50–53^.

Third, in addition to marital status and income, the scholastic grade of the children and adolescents was also significant. The majority were in the 8–11-year-old age group. The distribution of gender of the children was nearly equal (girls = 49.0%, boys = 51.0%). The participants with children attending grades 3 to 6 (usually including children in the 8–11-year-old age group) reported higher SCARED scores than other groups. One possibility for this is that at this age, children would likely be going to school under normal circumstances and therefore, the lockdown has limited their interactions with their peers. There is vast psychological literature suggesting that children of this age tend to develop social behaviors by interacting with their peers, rather than with their parents^54^. It is plausible that separation from their peers, as well as lack of access to social settings like playgrounds, have rendered these children vulnerable to increased anxiety and loneliness. Therefore, youth at this grade level may experience a greater interruption to their daily social life, causing more psychological distress^12,55^.

Thus, the aforementioned factors associated with higher SCARED scores in children and adolescents include living in divorced households or with low family income. The age group is another factor that contributed to the development of higher SCARED scores. The present identification of socio-demographic factors contributing to the development of anxiety symptoms has the potential to lay the groundwork for contemplating the prevention of poor mental health outcomes during and after the period of the COVID-19 pandemic.

### Limitations

These types of studies are prone to many limitations, and the most notable ones will be highlighted here. *First*, the generalization of this study is hampered by the fact that it was an online survey rather than a traditional pen-and-paper format. It is not clear whether the response rate tends to be higher among those who are ‘technologically savvy’, and might exclude parents who are not as technologically proficient. Oman has a high penetration of internet access, which has increased significantly in tandem with the onset of the pandemic and a resultant shift to online learning and working from home^56^. *Second*, this study aims for the nationally representative parent of the school-going population. The bulk of Oman's population is under the age of 20 years, and the present sample might fall short of the required national representative. This is a significant limitation of this study until large community surveys are conducted. *Third*, the study is likely to be limited by the fact that the parent version of the SCARED scale was used. It is not clear whether such a proxy approach adequately reflects the spectrum of anxiety-related symptoms among children and adolescents. Further study of the reliability of this version of the questionnaire is therefore warranted. In another study utilizing the Arabic version of the SCARED questionnaire, it was found to have a parallel capacity to the original English version of identifying the presence of anxiety-related symptoms^27^. In this study, both Arabic and English versions of the P-SCARED-41 were used. Future studies could examine whether different language versions of the P-SCARED-41 report similar results. *Fourth*, the cross-sectional design of this study provides a limited view of the longitudinal course of the mental well-being of the study’s participants. It is not clear whether the observed anxiety symptoms constitute acute stress reaction adjustment disorder or full-fledged anxiety symptoms with all the negative repercussions this is likely to have. *Last* but not least, while SCARED has been molded to follow the criteria of DSM, soliciting emotional symptoms via an online questionnaire is considered suboptimal compared to gold-standard structured interviews particularly tailored to non-western populations, where there is likely to be culture-specific idioms for distress and anxiety-related symptoms^31^.

## Conclusion

This study explores the frequency of anxiety-related symptoms in children and adolescents during the COVID-19 pandemic. Using the Screen for Child Anxiety Related Emotional Disorders (SCARED) for parents with a cut-off of > 25, one-third of the parents endorsed their children or adolescents to be marked with anxiety-related symptoms. The second aim was to explore the factors associated with anxiety-related symptoms in children. The notable risk factors were having a family income corresponding to low socio-economic status, having divorced or separated parents, and being in middle-grade levels in school. If this study were to withstand scrutiny, children could be appropriately directed toward available mental health services. The identification of risk factors and formulation of psychological interventions for vulnerable groups can significantly help mitigate the negative mental health impacts of the pandemic on the youth of the country.

## Data Availability

Access to data can be obtained by application to the Postgraduate Studies & Research (http://www.squ.edu.om/dor), College of Medicine and Health Sciences, Sultan Qaboos University. medpgsr@squ.edu.om.

## Abbreviations

GCC: Gulf cooperation council
OR: Odds ratio
SCARED: *Screen for child anxiety related emotional disorders*
USD: US dollar
WHO: World Health Organization
